# NANOG Expression as a Responsive Biomarker during Treatment with Hedgehog Signal Inhibitor in Acute Myeloid Leukemia

**DOI:** 10.3390/ijms18030486

**Published:** 2017-02-24

**Authors:** Seiji Kakiuchi, Yosuke Minami, Yoshiharu Miyata, Yu Mizutani, Hideaki Goto, Shinichiro Kawamoto, Kimikazu Yakushijin, Keiji Kurata, Hiroshi Matsuoka, Hironobu Minami

**Affiliations:** 1 Department of Medical Oncology and Hematology, Kobe University Graduate School of Medicine, Kobe 650-0017, Japan; kakiuchi@med.kobe-u.ac.jp (S.K.); yhmiyata@med.kobe-u.ac.jp (Y.M.); mizuyu@med.kobe-u.ac.jp (Y.M.); hgoto@med.kobe-u.ac.jp (H.G.); kkurata@med.kobe-u.ac.jp (K.K.); matsuoh@med.kobe-u.ac.jp (H.M.); hminami@med.kobe-u.ac.jp (H.M.); 2 Department of Transfusion Medicine and Cell Therapy, Kobe University Hospital, Kobe 650-0017, Japan; 3 Department of Medical Oncology and Hematology, Kobe University Hospital, Kobe 650-0017, Japan; skawamo@med.kobe-u.ac.jp (S.K.); kyakushi@med.kobe-u.ac.jp (K.Y.)

**Keywords:** hedgehog inhibitor, NANOG, acute myeloid leukemia, self-renewal, biomarker

## Abstract

Aberrant activation of the Hedgehog (Hh) signaling pathway is involved in the maintenance of leukemic stem cell (LSCs) populations. PF-0444913 (PF-913) is a novel inhibitor that selectively targets Smoothened (SMO), which regulates the Hh pathway. Treatment with PF-913 has shown promising results in an early phase study of acute myeloid leukemia (AML). However, a detailed mode of action for PF-913 and relevant biomarkers remain to be elucidated. In this study, we examined bone marrow samples derived from AML patients under PF-913 monotherapy. Gene set enrichment analysis (GSEA) revealed that PF-913 treatment affected the self-renewal signature and cell-cycle regulation associated with LSC-like properties. We then focused on the expression of a pluripotency factor, NANOG, because previous reports showed that a downstream effector in the Hh pathway, GLI, directly binds to the NANOG promoter and that the GLI-NANOG axis promotes stemness and growth in several cancers. In this study, we found that a change in NANOG transcripts was closely associated with GLI-target genes and NANOG transcripts can be a responsive biomarker during PF-913 therapy. Additionally, the treatment of AML with PF-913 holds promise, possibly through inducing quiescent leukemia stem cells toward cell cycling.

## 1. Introduction

The Hedgehog (Hh) signaling pathway is linked to tumorigenesis and is aberrantly activated in a variety of cancers [1,2]. Hh ligands bind to and suppress the transmembrane receptor Patched (PTCH), which suppresses Smoothened (SMO), a seven-transmembrane-helix protein that positively regulates the Hh pathway [3]. When SMO is activated, glioma-associated oncoproteins (GLI1, GLI2, GLI3) translocate to the nucleus and subsequently activate the downstream Hh signaling pathway, which promotes the transcription of target genes (Figure S1). Intriguingly, Hh signaling components are reported to be expressed in acute myeloid leukemia (AML) cells [4,5]. The activity of the Hh signaling pathway is thought to be required for the maintenance of a leukemia stem cell (LSC) population at least in some experimental systems [5,6,7]. Interestingly, the eradication of these cells may be essential for reducing the recurrence of disease, implying that targeting the Hh signaling pathway may be the treatment of choice in a permanent cure for AML [8,9]. In particular, elderly AML/MRC (AML with myelodysplasia-related changes) patients without molecular targets induced by strong driver gene mutations, such as FLT3 (Fms-like tyrosine kinase 3) and IDH2 (isocitrate dehydrogenase 2), continue to have unmet medical therapeutic needs [10]. PF-0444913 (PF-913) is a novel oral small molecule inhibitor that selectively binds and targets SMO [11]. Treatment with PF-913 has shown promising results in terms of safety, tolerability, and initial signs of efficacy in an early phase study of hematologic malignancies, including AML [11]. However, a detailed mode of action and the identification of therapeutic biomarkers involved with Hh pathway inhibitors remain to be elucidated in AML therapy. In this article, we show NANOG transcript level can be a responsive biomarker during treatment with PF-913 in AML. Transcription of NANOG, one of a pluripotency factor, is known to be located downstream of GLI and activated by GLI proteins in several cancer models [12,13].

## 2. Results

### 2.1. Gene Expression Profiling from AML Patients during Treatment with PF-913 Monotherapy

**Figure 1:**
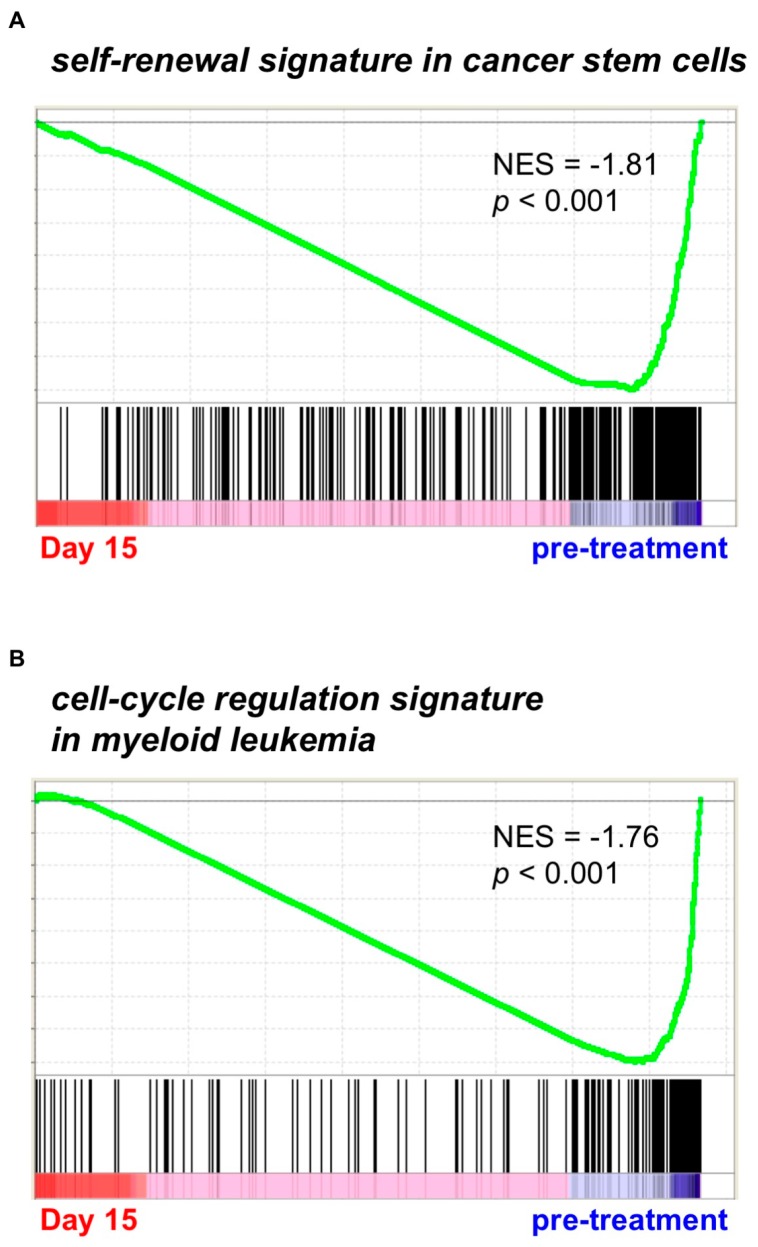
PF-913 treatment of clinical AML patients commonly affected self-renewal and cell-cycle statuses Gene Set Enrichment Analysis (GSEA) showed that (**A**) the self-renewal signature was highly modulated during PF-913 treatment in one AML patient, while (**B**) the cell-cycle signature was modulated in all patients. NES: normalized enrichment scores.

Using bone marrow leukemia cells derived from three AML/MRC patients under PF-913 monotherapy, we conducted GSEA. This revealed that a bone marrow sample from one of the three patients showed a change in self-renewal signature, namely cancer stem cell-like gene signature (Figure 1A) [14]. Furthermore, bone marrow samples from all three AML patients revealed a change in a cell-cycle regulation signature, such as that of myeloid leukemia cell-cycle activity (Figure 1B) [15]. We therefore immunostained for Ki-67, which revealed that AML cells taken from patients on Day 5 of PF-913 treatment stained more strongly than control cells; however, the proportion of stained cells decreased in samples taken at Weeks 2 and 4 as shown in Figure S2.

### 2.2. PF-913 Treatment Dysregulate NANOG Expression In Vivo

**Figure 2:**
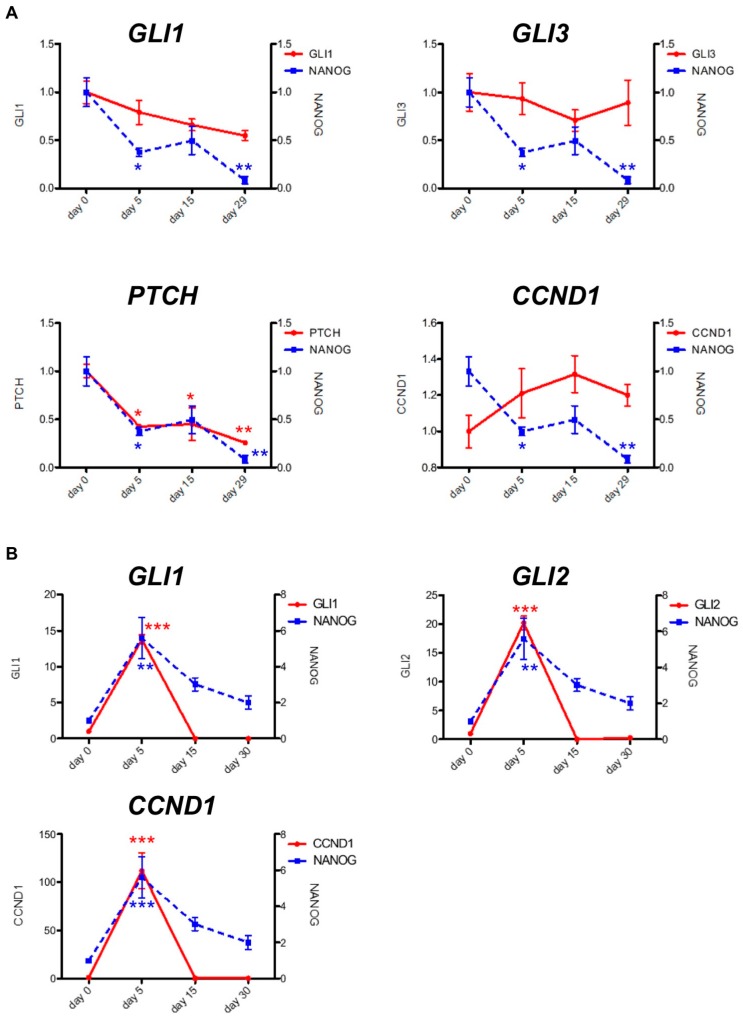
Changes in NANOG transcript levels were coincident with GLI-target genes. Transitions in NANOG and GLI-target gene transcript levels were associated together in (**A**) one AML patient under clinical PF-913 monotherapy, as well as in (**B**) another AML patient. Data are expressed as the mean ± SEM. The significance of differences between groups was analyzed using the Bonferroni test, followed by one-way analysis of variance (ANOVA). Statistical analyses were performed with GraphPad Prism. * *p* < 0.05, ** *p* < 0.01, *** *p* < 0.001.

We also noted that a self-renewal signature ranked highly in samples from AML patients undergoing PF-913 monotherapy. We examined NANOG expression as a self-renewal biomarker using qPCR on bone marrow samples from three AML patients (Patient 1, 2, and 3) undergoing PF-913 monotherapy. One patient’s sample revealed that transcript levels of GLI-target genes, such as GLI1 and PTCH, decreased while the NANOG transcript levels progressively diminished (Figure 2A). The NANOG transcript levels also correlated with GLI-target genes including a temporarily increase on Day 5 (Figure 2B). Although the expression level of CCND1, the cell cycle-related molecule, did not decrease, gene profile analysis showed that some cell-cycle associated molecules were downregulated comparing the samples of 2 weeks after treatment with those of pre-treatment among three patients. (Figure S3). We also immunostained GLI1 and NANOG proteins in bone marrow samples from PF-913 monotherapy patients (Figure S4). In the clinical trial, the transitions of white blood cell differentiation in three patients are shown in Figure S5.

### 2.3. Pharmacological and Genetic Knockdown of SMO Lead to Decrease NANOG Expression In Vitro Model

**Figure 3:**
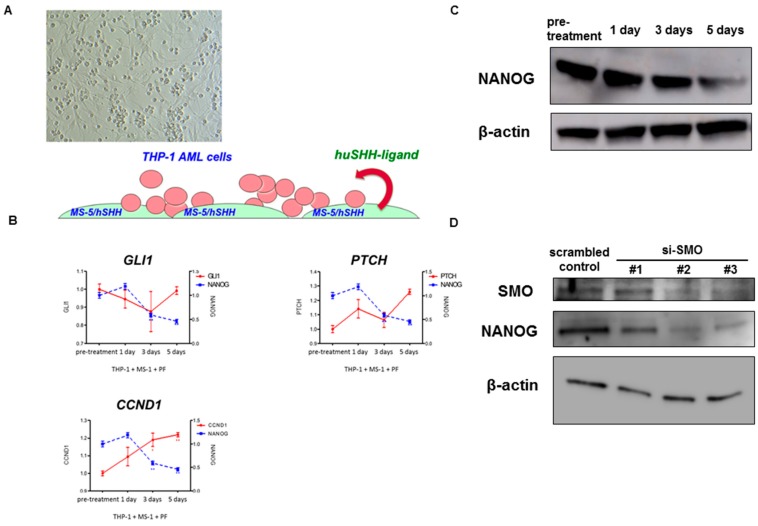
Inhibiting SMO pharmacologically and genetically leads to dysregulation of NANOG expression in vitro model. (**A**) A co-culture model of an AML cell line (THP-1) with stroma which overexpresses human sonic hedgehog (SHH) ligands; (**B**) NANOG transcripts substantially decreased during PF-913 treatment in THP-1 cells co-cultured with MS-5 cells over-expressing human SHH ligands. In a pre-clinical setting, although the level of GLI-target gene transcript did not decrease much, that of NANOG decreased during the time course; (**C**) Western blotting showed the NANOG protein level decreased during PF-913 treatment; (**D**) Western blot analysis showed downregulated NANOG protein after transfection with SMO siRNAs in comparison to cells transfected with scrambled control. Although knockdown effect of SMO by si-SMO#1 was weak compared with #2 or #3, NANOG expression was evident as well.

Due to a decrease in NANOG transcript levels in PF-913 monotherapy patient cases, we reverted to a pre-clinical setting using a co-culture model of an AML cell line with stroma, which overexpressed human sonic hedgehog ligands (Figure 3A). In this model, although GLI-target gene transcript levels did not change greatly, the NANOG transcript level progressively decreased over time (Figure 3B). In the same pre-clinical model, PF-913 treatment also decreased the expression of NANOG protein levels as determined by Western blot (Figure 3C). We also used siRNAs targeting SMO instead of treatment with PF-913, they also decreased the NANOG expression level in Western blotting. (Figure 3D).

## 3. Materials and Methods

### 3.1. DNA Microarray and Data Analysis

Total RNA was purified from bone marrow cells of patients undergoing treatment with PF-913. DNA microarray analysis was taken on by Filgen (Aichi, Japan) using a GeneChip Human Gene 2.0 ST Array (Applied Microarrays, Tempe, AZ, USA).

### 3.2. Gene Set Enrichment Analysis

Gene set enrichment analysis (GSEA) was carried out to interpret microarray data affected by PF-913. We used a GSEA algorithm to evaluate the statistical significance of gene expression. GSEA is mounted by the Broad Institute of MIT and Harvard website (http://www.broadinstitute.org/gsea/index.jsp). GSEA calculates normalized enrichment scores (NES), which are values assigned to each gene and are set after normalization across all analyzed gene sets. To estimate the statistical significance of NES, nominal *p*-values and false discovery rates (FDR) are usually used. We used a criteria of a nominal *p*-value < 5% and a FDR < 25% as the statistical cutoff for all analyses.

### 3.3. Real-Time RT-PCR

Total RNA was purified using a NucleoSpin RNA kit (Macherey-Nagel, Duren, Germany), and reverse transcription was performed using a PrimeScript RT Reagent Kit (Roche, Basel, Switzerland). We performed real-time RT-PCR according to standard procedures using a KOD SYBR qPCR Mix (Toyobo, Osaka, Japan), quantitative PCR primers for GLI1, GLI2, GLI3, PTCH1, Cyclin D1, NANOG, and a LightCycler 480 System PCR platform (Roche). Primer sequences used were CCT TGG AAG GTG ATA TGT CCA G (forward) and AGC TGC TCT TGG GAG TCA AA (reverse) for GLI1; GAC ATT CGG CTA AGG AGG GAT T (forward) and CCA AAT GCT CCC TAC CAT CTT TC (reverse) for GLI2; AGG CTG AAC CTA AGC TCT GTT G (forward) and CGT ACA CTG TCC ATA TCA CCA CTC (reverse) for GLI3; CTG CTG GGC CTC GTA GTG CCG AAG C (forward) and CTG TTG GCA TAG GAG TGG AGT TCA CC (reverse) for PTCH1; CCT CGG TGT CCT ACT TCA AAT G (forward) and TGG TCT CCT TCA TCT TAG AGG C (reverse) for Cyclin D1; CCT GTG ATT TGT GGG CCT G (forward) and GAC AGT CTC CGT GTG TGA GGC AT (reverse) for NANOG; and GGA CTT CGA GCA AGA GAT GG (forward) and AGC ACT GTG TTG GCG TAC AG (reverse) for β-actin. Results were normalized relative to β-actin expression. The relative levels of mRNA were calculated using the 2^−(ΔΔ*C*t)^ method.

### 3.4. Statistical Analysis

Data are expressed as the mean ± SEM. The significance of differences between groups was analyzed using the Bonferroni test, followed by one-way analysis of variance (ANOVA). Statistical analyses were performed using GraphPad Prism 5.0. (GraphPad Software Inc., La Jolla, CA, USA).

### 3.5. Reagents

PF-913 was supplied by Pfizer (La Jolla, CA, USA), and stored at −20 °C as a 10^−2^ M stock solution in DMSO (dimethyl sulfoxide) for in vitro experiments.

### 3.6. Cells

MS-5 cells overexpressing human sonic hedgehog ligands were supplied by Pfizer. AML cell lines were cultured in RPMI 1640 Medium (Sigma, St. Louis, MO, USA) containing 10% fetal bovine serum (FBS) (Gibco-BRL, Grand Island, NY, USA), and MS-5 cells were cultured in Dulbecco’s Modified Eagle’s Medium containing 10% FBS. We plated an AML cell line (THP-1) with MS-5 cells, which stably overexpressed human sonic hedgehog (SHH) ligands, into four dishes. Cells were then co-cultured for five days in RPMI 1640 Medium containing 10% FBS, and each dish was treated with or without PF-913 (1 µM) for 24 h, 3 or 5 days. Human leukemic blasts were isolated from patients with AML after informed consent was obtained in accordance with the Declaration of Helsinki. The trial was approved by the institutional ethics committee of Kobe University (26 June 2014) and was registered on the University hospital Medical Information Network (UMIN) Clinical Trials Registry (UMIN000011798, 1 October 2013). Written informed consent was obtained from all participants.

### 3.7. Western Blotting and Immunohistochemistry

Antibodies against NANOG (ab80892) and β-actin (A1978) were purchased from Abcam (Cambridge, UK) and Sigma, respectively. Western blotting was performed using standard protocols as previously described [16,17]. Immunohistochemistry was performed on sections cut from bone marrow biopsy blocks of formalin-fixed, paraffin-embedded tissue at the Institute of Pathology (Kobe University Hospital, Kobe, Japan). Ki-67 antibody (IR626; Dako, Tokyo, Japan) was used undiluted. Anti-NANOG (ab62734; Abcam) and anti-GLI1 antibodies (H-300; Santa Cruz, Dallas, TX, USA) were diluted 1/75 and 1/2000, respectively. Stained cells were photographed using a ×10 objective lens on an HS All-in-one Fluorescence Microscope BZ-X710 (Keyence, Osaka, Japan), and then analyzed with BZ-X Analyzer software 1.3.0.3. (Keyence).

### 3.8. siRNA Transfection

For silencing the expression of SMO, THP-1 cells were transfected with siRNAs (#1: HS0215118; #2: HS0215119; #3: HS0215120) or scrambled negative control. (Takara Bio, Tsu, Japan) Transfection was done with Nucleofector Kit V. (Lonza, Bazel, Switzerland) SiRNA treated-cells were cultured for 48 h.

### 3.9. Patient Characteristics

We treated three patients in the clinical study with PF-913 monotherapy. Three patients were all diagnosed with AML/MRC without specific driver gene mutations such as FLT3.

Patient 1 (65 year-old female) was diagnosed with AML/MRC harboring complex karyotype. She underwent two courses of induction therapy; however, her disease did not attain response. Thereafter, she took one salvage regimen and did not improve. When she started the clinical trial, her laboratory data were as follows: WBC (white blood cell) 5900 /µL (Blast 42.0%), Hb 7.7 g/dL, Plt 50,000 /µL. During treatment, she often needed red blood cell or platelet transfusion. Her laboratory data changed as follows: after 1 course: WBC 28,900 /µL (Blast 60.0%), Hb 8.1 g/dL, Plt 17,000 /µL; after 2 courses: WBC 19,100 /µL (Blast 60.0%), Hb 9.3 g/dL, Plt 19,000 /µL; after 3 courses: WBC 12,300 /µL (Blast 67.0%), Hb 9.0 g/dL, Plt 9000 /µL, after 4 courses: WBC 24,300 /µL (Blast 66.0%), Hb 7.6 g/dL, Plt 25,000 /µL; after 5 courses: WBC 36,100 /µL (Blast 86.0%), Hb 6.4 g/dL, Plt 30,000 /µL. She died of intracranial hemorrhage suddenly during Course 6 and the laboratory data at that time were as follows: WBC 41,100 /µL (Blast 90.0%), Hb 8.4 g/dL, Plt 16,000 /µL.

Patient 2 (73-year-old male) was diagnosed with AML/MRC harboring chromosomal abnormality; 46XY, t(17;21) (q21;q22). Despite induction therapy, he could not attain hematological response. When he started the clinical trial, his laboratory data were as follows: WBC 5500 /µL (Blast 15.0%), Hb 7.0 g/dL, Plt 27,000 /µL. He also occasionally needed red blood cell or platelet transfusion. Her laboratory data changed as follows: after 1 course: WBC 16,800 /µL (Blast 44.0%), Hb 8.9 g/dL, Plt 61,000 /µL; after 2 courses: WBC 26,200 /µL (Blast 50.0%), Hb 7.1 g/dL, Plt 37,000 /µL; after 3 courses: WBC 44,600 /µL (Blast 44.0%), Hb 6.2 g/dL, Plt 43,000 /µL. He stopped treatment after Course 3 due to disease progression.

Patient 3 (72-year-old female) was diagnosed with AML/MRC harboring normal karyotype. She attained hematological complete remission after 2 courses of induction therapies and underwent 2 consolidation therapies. When she started clinical trials, her laboratory data were as follows: WBC 30,500 /µL (Blast 76.0%), Hb 9.6 g/dL, Plt 46,000 /µL. She did not need transfusion during clinical trial. Her laboratory data changed as follows: after 1 course: WBC 17,200 /µL (Blast 50.0%), Hb 10.0 g/dL, Plt 43,000 /µL. She stopped treatment after Course 1 due to disease progression.

## 4. Discussion

Our research implies that Hedgehog inhibitor PF-913 treatment can modulate self-renewal and cell-cycling status during treatment with AML. We investigated the effects of Hedgehog signaling inhibitors on signatures in primary AML cells using PF-913 monotherapy patients’ bone marrow samples. Conducting those samples with GSEA, it revealed that PF-913 treatment influenced self-renewal capacity and cell-cycling status. We particularly focused on self-renewal and cell-cycling signatures since we examined effects of in vivo treatment with PF-913 beforehand using an immunodeficient NOD/SCID/IL2rγnull (NOG) mice model [18]. In that experiment, NOG mice were injected with AML cells and treated with either a vehicle control or PF-913 for 10 days after engraftment of AML cells. Mouse bone marrow was harvested, and AML cells were analyzed on human CD45 and sorted; thereafter, we examined gene expression profiling. From the experiment, we found that self-renewal and cell-cycling signatures were highly expressed in both signatures with NOG mice and AML patients (data not shown).

According to the in vivo-treatment data with NOG mice and clinical patients’ sample, it was revealed PF-913 affected cell-cycling status. We confirmed the cell-cycling status was modulated through PF-913 treatment by using Ki-67 immunostaining of bone marrow samples from AML patients. Hence, immunostained area with Ki-67 transiently increased in an early stage, we hypothesized that PF-913 treatment possibly induced cell-cycling in dormant cells. Gene expression profile also showed that some cell cycle-related molecules were decreased during PF-913 treatment in each patient.

As the gene expression profile revealed self-renewal capacity was also modulated commonly in NOG mice and AML patients, we checked the NANOG transcript level. We focused on NANOG expression as an example of a self-renewal-associated molecule because NANOG, one of the representative pluripotency factor, is a downstream effector in the Hh pathway. GLI1 and GLI2 directly bind to the NANOG promoter, with the GLI-Nanog axis promoting stemness and growth in several cancers [12,13]. We found that NANOG transcript levels in PF-913 monotherapy patient cases; furthermore, the immunostained area with NANOG protein diminished during treatment. We also found white blood cell differentiation during treatment. The proportion of segmented cells transiently increased after PF-913 treatment in Patients 1 and 2, who had long-term treatment with PF-913.

Since the in vivo experiments using PF-913 monotherapy patients’ samples showed us the treatment with PF-913 dysregulated NANOG, we returned to the pre-clinical setting. The in vivo experiments also revealed that PF-913 treatment decreased the expression of NANOG both in mRNA and protein levels. We genetically knocked down SMO using siRNA and obtained the same result in Western blot analysis.

In this study, we showed NANOG expression is useful as a responsive biomarker because NANOG decreased stringently during treatment. NANOG is known to be a key molecule in regulating glioma stem cells and is located downstream of the hedgehog signaling pathway [12]. However, NANOG has never been reported to have a correlation with GLI-NANOG axis in AML. We here for the first time show that there is a correlation in AML by using PF-913 in a clinical study as well as a pre-clinical model. 

In addition, it is noteworthy that GSEA showed that PF-913 modulates self-renewal signature. From this result, we consider inhibiting the hedgehog signaling pathway can be an effective treatment option owing to its influence to self-renewal status in treating with AML. Conventional chemotherapy can eliminate highly proliferative tumor burden, while sparing the relatively dormant cells and these cells lead to resistance to chemotherapy [19]. Therefore, targeting the dormant leukemic stem cells is a critical problem to overcoming the resistance. Hence, we showed that Hedgehog signaling inhibitor PF-913 acts on the stemness and that this therapy can be a novel strategy. Recently, it was reported that PF-913 monotherapy is tolerant to, and safe for, refractory or relapsed AML in Phase I clinical trials [11]. However, it has also been shown that PF-913 monotherapy is not efficient to control the disease. Here we show that PF-913 can possibly induce dormant cells into the cell cycle by using Ki-67 immunostaining. Therefore, PF-913 plus other therapy can be a promising strategy as an AML treatment; for example, PF-913 plus conventional chemotherapy or novel therapy including molecular target therapy and immune check point inhibitors. It remains unknown what drugs are the most effective to use as a combination therapy with PF-913 or what concrete changes invoke in leukemic stem cells, so these topics need to be explored further in future studies. There is increasing evidence that the Hh pathway is targeted in hematological malignancy, and many novel drugs targeting the Hh pathway, inhibiting not only SMO but also Hh ligand or GLI, are available [20,21]. Many clinical trials involving Hh inhibitors monotherapy or combination therapy are ongoing in hematological malignancies [22]. Furthermore, it became apparent that drugs usually used in hospital practice like itraconazole or arsenic trioxide have anti-Hh signaling activity [23,24]. As dietary therapy, sulphoraphane or the combination of epigallocatechin-3 gallate and quercetin is reported to inhibit the self-renewal activity via Hh pathway in pancreatic cancer [25,26].

In conclusion, gene profiling analyses revealed that treatment with the Hh signaling inhibitor, PF-913, modulates self-renewal and cell-cycle signatures in AML. The NANOG transcript level was also shown to be a responsive biomarker during PF-913 therapy. By promoting quiescent LSCs toward a cell-cycle status, PF-913 therapy appears promising as a treatment for AML. This is the first report that illustrates that PF-913 treatment modulates the self-renewal status and specifically clarifies NANOG can be a responsive biomarker in AML.

## Supplementary Materials

Supplementary materials can be found at www.mdpi.com/1422-0067/18/3/486/s1.
