# Supplementary material for: NANOG Expression as a Responsive Biomarker during Treatment with Hedgehog Signal Inhibitor in Acute Myeloid Leukemia
Source: Int J Mol Sci. 2017 Feb 24;18(3):486. doi: 10.3390/ijms18030486 (PMC5372502; doi:10.3390/ijms18030486)
Supplement: Supplementary file 1 [file ijms-18-00486-s001.pdf]

Supplementary Material: NANOG Expression as a Responsive Biomarker during Treatment with Hedgehog Signal Inhibitor in Acute Myeloid Leukemia

Seiji Kakiuchi, Yosuke Minami, Yoshiharu Miyata, Yu Mizutani, Hideaki Goto, Shinichiro Kawamoto, Kimikazu Yakushijin, Keiji Kurata, Hiroshi Matsuoka and Hironobu Minami

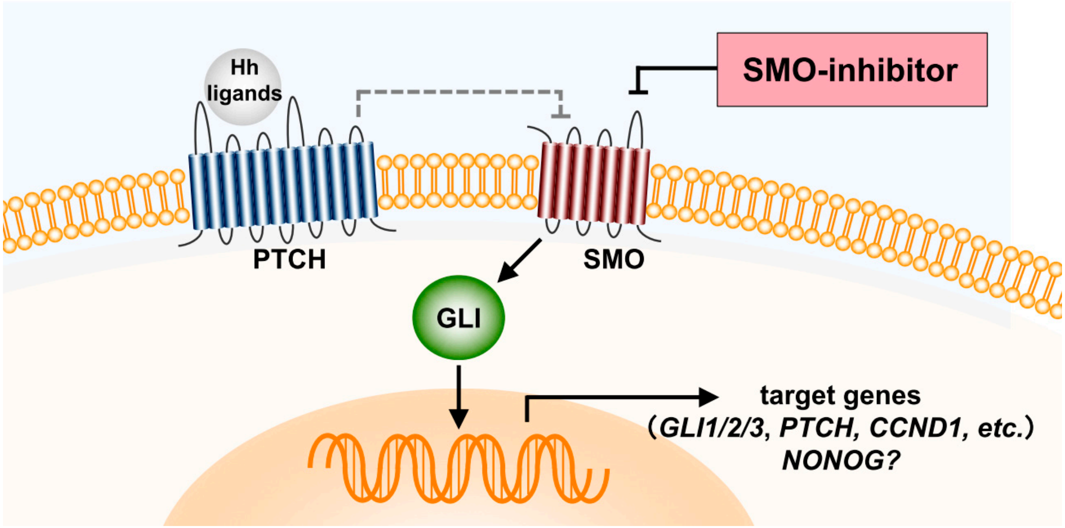

Figure S1. A schema of the Hh signaling pathway and smoothened (SMO) inhibitor.

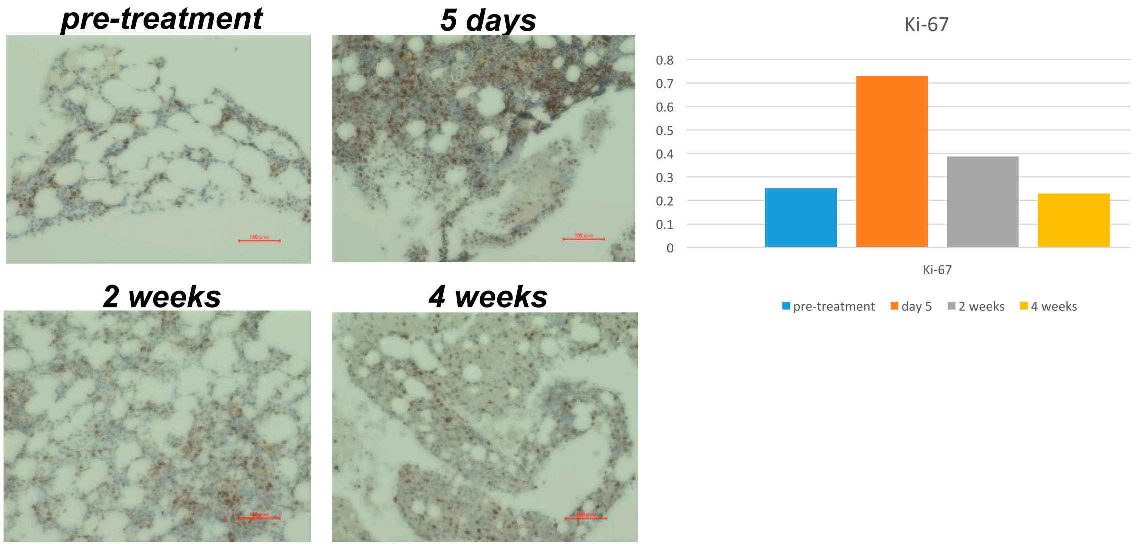

Figure S2. Ki-67 immunostaining of bone marrow samples derived from AML patients in treatment with PF-913. Stained cells were photographed using a 10× objective lens on an HS All-in-one Fluorescence Microscope BZ-X710 (Keyence, Osaka, Japan), and then analyzed with BZ-X Analyzer software 1.3.0.3 (Keyence). Scale bar indicates 100 µm. AML cells taken from patients on day 5 were stained more strongly than control cells. The proportion of stained cells within samples decreased on week 2 and 4.

a

Gene Description	Expression value {Day 15}	Expression value {Day 0}	Ratio
cyclin-dependent kinase 6	546.7675	690.3224	0.7920466
cyclin-dependent kinase 17	258.5784	326.6813	0.7915311
cyclin A1	132.0147	168.6279	0.7828758
cyclin K	241.9618	314.6301	0.7690358
cyclin D binding myb-like transcription factor 1	456.7834	639.9221	0.7138109
cyclin-dependent kinase-like 2 (CDC2-related kinase)	11.16589	16.88885	0.6611397

b

Gene Description	Expression value {day 15}	Expression value {day 0}	Ratio
cyclin B2	79.40307	99.57973	0.7973819
cyclin F	73.74847	94.77658	0.7781297
cyclin-dependent kinase 8	74.08309	96.19574	0.7701286
cyclin-dependent kinase inhibitor 2C	27.17594	36.00911	0.7546962
cyclin D-type binding-protein 1	134.4164	185.2599	0.7255558
cyclin C	31.47397	44.08308	0.7139694
cyclin E1	72.57008	102.3358	0.7091368
cyclin A2	226.0831	345.4463	0.6544667
cyclin B1	60.95027	94.3652	0.6458977
cyclin-dependent kinase inhibitor 3	81.78414	132.761	0.6160253
cyclin-dependent kinase 1	78.22661	155.9832	0.5015066

c

Gene Description	Expression value {day 15}	Expression value {day 0}	Ratio
cyclin-dependent kinase 2 interacting protein	32.69574	42.3108	0.7727516
cyclin O	12.18868	15.79771	0.7715473
cyclin-dependent kinase 1	118.5653	154.0527	0.7696412
cyclin-dependent kinase inhibitor 3	79.39215	105.1104	0.7553215
cyclin-dependent kinase-like 5	12.32727	16.62435	0.7415189
cyclin B2	141.8313	203.3475	0.6974824
cyclin-dependent kinase 11A // cyclin-dependent kinase 11B	54.31515	82.37109	0.6593958
cyclin M2	71.35423	121.1865	0.5887969
cyclin E1	134.8587	239.6956	0.5626248
cyclin F	66.79519	120.1149	0.5560941
cyclin D-type binding-protein 1	163.2991	306.0949	0.5334917
cyclin-dependent kinase 14	42.20995	86.80894	0.4862397

Figure S3. Gene profile analysis showed us that some of cell-cycle associated molecules were down-regulated comparing the samples of 2 weeks after treatment with those of pre-treatment among three patients.

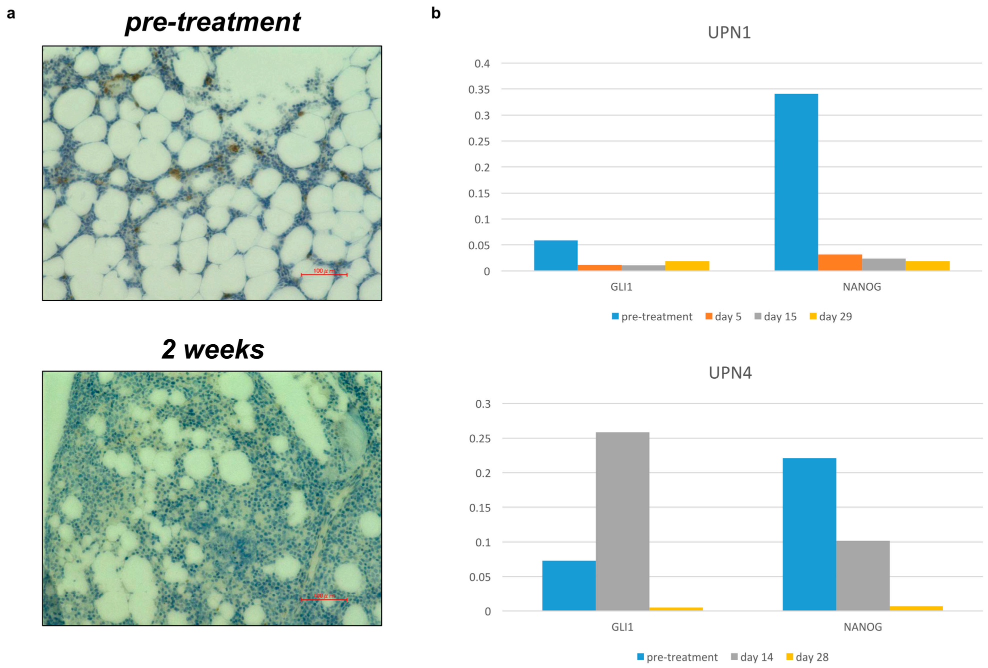

Figure S4. Immunostaining of patients' bone marrow samples with NANOG antibody. The proportions of stained area decreased during treatment with PF-913. Scale bar indicates 10 μm.

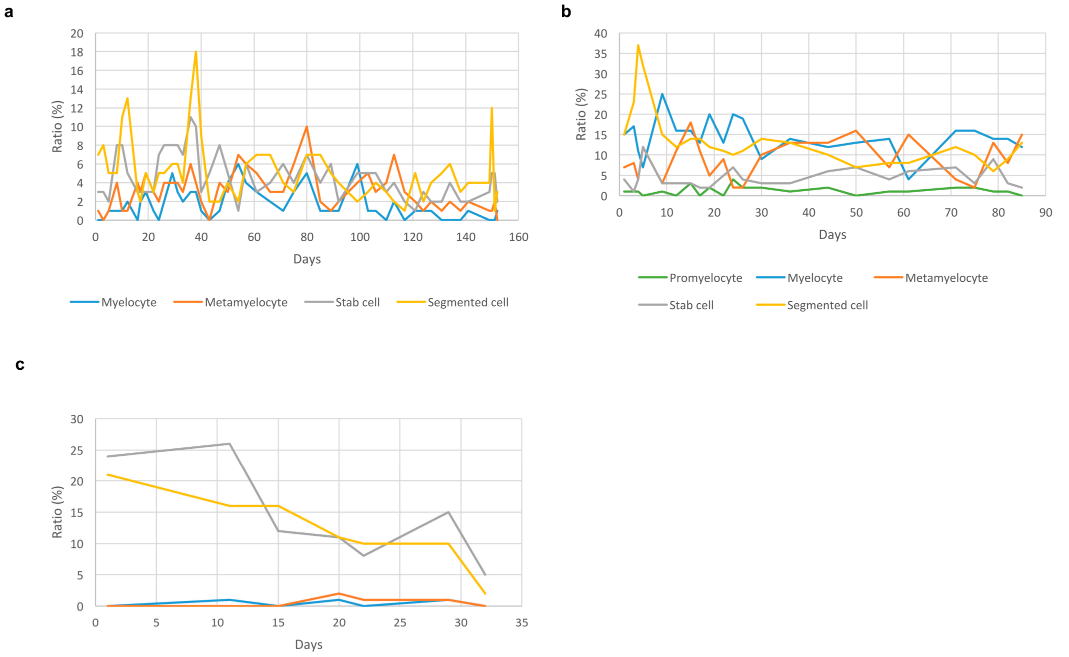

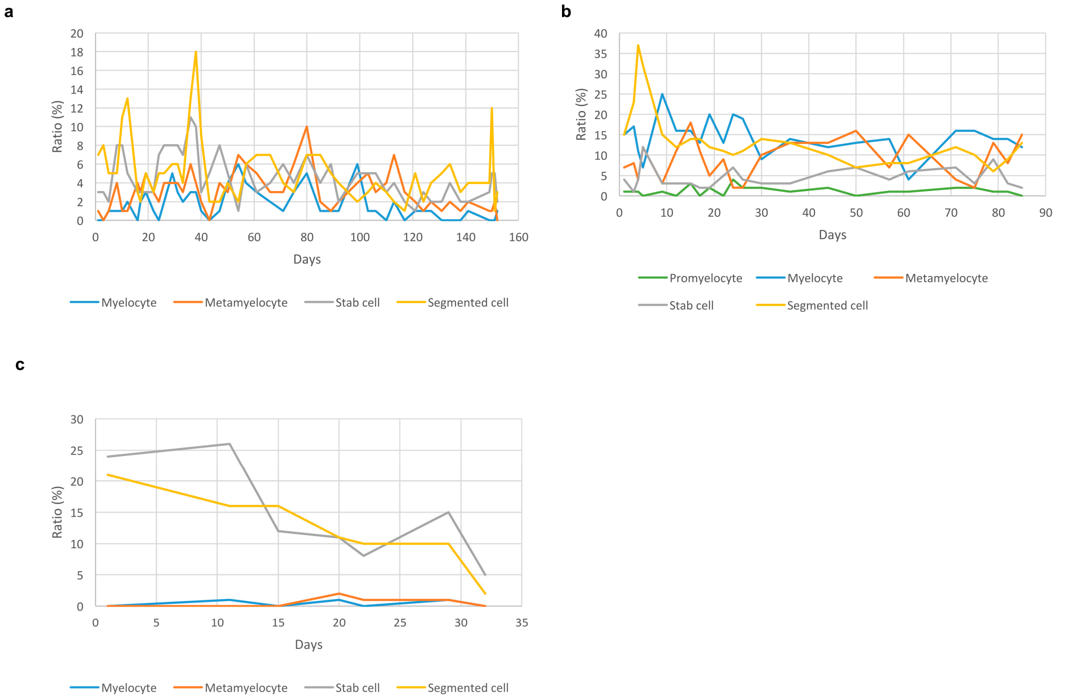

Figure S5. WBC data during treatment with PF-913. The transitions of white blood cell differentiation in three patients.
